# 

**Published:** 1886-12

**Authors:** 


*American Dental Association*—Meets on the 1st Tuesday of August, 1886. Place to be fixed by the Executive Committee. Pres. W. C. Barrett, Buffalo, N. Y.; Cor. Sec. A. W. Harlan, Chicago; Rec. Sec. Geo. H. Cushing, Chicago.

### STATE DENTAL SOCIETIES.

*Alabama Dental Association*—Meets annually. Next meeting at Montgomery, on the second Tuesday of April, 1886. Pres. W. R. McWilliams, Athens; Sec. C. A. Merrill, Birmingham.

*California State Dental Association*—Tuesday, May 22d, 1886. Pres. Wm. Dutch, San Francisco; Sec. S. E. Goe., San Francisco; Cor. Sec. H. E. Knox, San Francisco.

*Connecticut State Dental Society*—Pres. R. W. Brown, New London; Rec. Sec. Geo. L. Parmelee, Hartford. Annual meeting third Tuesday in May, 1886, at Hartford.

*Dental Society of the State of Maryland and District of Columbia*—Meets annually. Next meeting at Washington City, on the second Tuesday in October, 1886. Pres. B. F. Coy, of Baltimore: Sec. M. W. Foster, M. D.

*Georgia State Dental Society*—Meets second Tuesday in May, 1886, at Savannah, Ga. Pres A. G. Boynton, Savannah; Rec. Sec. G. W. H. Whitaker, Sandersville; Cor. Sec. L. D. Carpenter, Atlanta.

*Illinois State Dental Society*—Meets annually. Pres. T. L. Gilman, Quincy; Sec. J. W. Wassel, Chicago. Next place of meeting, Rock Island second Tuesday in May, 1886.

*Indiana State Dental Society*—Meets next on the last Tuesday of June, 1886. Pres. S. B. Brown, Ft. Wayne; Sec. Robert W. Van Valzah, Terre Haute.

*Iowa State Dental Society*—Meets annually. Next meeting in Iowa City, on the first Tuesday in May, 1886. Pres. A. Morsman, Iowa City; Rec. Sec. J. B. Montfort, Fairfield.

*Kentucky State Dental Association*—Meets annually, first Tuesday in June, 1886. Next meeting in Louisville. Pres. J. M. Clyde, Covington; Sec. E. C. Dunn, Louisville.

*Kansas State Dental Association*—Pres. L. C. Wasson, Ottawa; Sec. C. B. Reed, Topeka. Next meeting will be held at Topeka, first Tuesday in May, 1886.

*Maine Dental Society*—Meets in August, at Thomaston. Pres. E. J. Robert, Augusta, Sec. C. E. Hussey, Beddeford.

*Maryland State Dental Association*—Meets annually next November 13. Pres. T. J. Smithers; Rec. Sec. Wm. A. Mills; Rec. Sec. B. M. Hopkinson.

*Massachusetts Dental Society*—Meets annually in Boston, second Thursday in December. Pres. F. Searl, Springfield; Rec. and Cor. Sec. W. E. Page, Boston.

*Michigan State Dental Association*—Meets annually. Next meeting at Ann Arbor, last Wednesday in March, 1886. Pres. J. Lathrop, Detroit; Sec. J. B. McGregor, Port Huron; Treas. H. K. Lathrop, Detroit.

*Mississippi State Dental Association*—Will meet August 4, 1886, at Jackson Pres. E. E. Spinks, Meridian; Sec. W. W. Westmorelad, Columbus.

*Minnesota Dental Association*—Meets annually. Meets May 26, 1886, at Winona. Pres. F. A. Williamson, Red Wing; Sec. M. G. Jenison, Minneapolis.

*Missouri Dental Association*- Meets annually on the first Tuesday of July, 1886, Next meeting at Sweet Springs. Pres. A. J. Prosser, St. Louis; Sec. Geo. L. Shepard, Sedalia.

*Nebraska State Dental Society*—Meets annually. Next meeting third Tuesday in May 1886, at Beatrice; Pres. J. J. Willey, Wahoo; Sec. C. R. Tefft, Lincoln.

*New Jersey State Dental Society*—Meets annually. Next meeting at Long Branch. on Wednesday, July, 20th 1885. Pres. Dr. J, W. Scarborough, of Lambbertville; Sec. C. A. Meeker, Newark.

*New Hampshire Dental Society*—Pres. D. W. Eagerly, Farmington; Sec. C. W. Clerment [illegible]anchester.

*North Carolina State Dental Society*—Meets annually. Next meeting at Raleigh, first Tuesday in June, 1886. Pres. J. E. Mathews, Kenansville; Sec. Thos. M. Hunter, Fayetteville.

*Ohio State Dental Society*—Meets annually. Next meeting at Toledo, last Tuesday of October, 1886. Pres. F H. Rehwinkle, Chillicothe; Sec. J. R. Callahan, Hillsboro.

*Oregon State Dental Society*—Meets annually. Pres. J. R. Cardwell, Portland; Rec. Sec. S. J. Barber.

*Pennsylvania State Dental Society*—Meets Tuesday, July 27th, 1886, at Cresson Springs; Pres. E. H. Neal, Philadelphia; Rec. and Cor. Sec. Th. F. Chupein, Philadelphia.

*Rhode Island Dental Association*—Pres. A. W. Buckland, Woonsocket; Sec. L. L. Buckland, Providence.

*South Carolina State Dental Association*—Meets annually. Pres. A. P. Johnston, Anderson; Sec. G. F. S. Wright, Columbia; Rec. Cor. Sec. R. A. Smith, Charleston. Next meeting at Cheraw, Tuesday, June 1, 1886.

*Texas State Dental Association*—Meets in the city of Austin, May 4, 1886. Pres. S. E. Jones, Houston. Sec. G. M. Patten, Huntsville.

*Tennessee Dental Association*—Meets Annually. Next meeting at Nashville third Tuesday in February, 1886. Pres. J. W. Lee, Chattanooga; Rec. Sec. D. B. Blakemore, Nashville; Cor. Sec. R. B. Lees, Nashville.

*The Dental Society of the State of New York*—Meets annually on the second Wednesday in May. Next session at Albany, May 12, 1886. Pres. Frank B. Darly, Elmira; Rec. Sec. J. Ewd. Line, Rochester; Cor. Sec. W. H. Atkinson, New York.

*Vermont State Dental Society*—Meets annually. Next meeting at Bellows Falls, March 17, 1886. Pres. G. W. Swift, Bethel; Sec. Thos. Mound, Rutland.

*Virginia State Dental Association*—Meets in Charlottsville, Tuesday, August 19, 1885. Pres. J. Hall Moore; Sec. L. M. Cowarden, Richmond.

*Wisconsin State Dental Society*—Meets annually. Next meeting at Milwaukee, July 20, 1886. Pres. B. G. Marklein, Milwaukee; Sec. C. A. Southwell, Appleton, Wis.

### LOCAL SOCIETIES.

*American Academy of Dental Science*—Meets annually in Boston, Mass., on the last Wednesday in October, 1885. Pres. Geo. T. Moffatt; Rec. Sec. E. E. Hopkins; Cor. Sec. E. B. Hitchcock, Boston.

*Brooklyn Dental Society and Second District Society United*—Meets quarterly—Pres. T. H. Holly; Rec. Sec. A. N. Russell, Brooklyn.

*Central Dental Association of Northern New Jersey*—Meets annually in February. Pres. C. A. Meeker, Newark, Sec. G. Carleton Brown, Elizabeth.

*Central Illinois Dental Society*—Meets annually. Next meeting at Bloomington Oct. 13, 1885; Pres. J. D. Moody, Mendota; Sec. C. R. Taylor, Streetor.

*Chicago Dental Society*—Meets monthly. C. F. Matteson; Sec. A. W. Hoyte.

*Connecticut Valley Dental Society*—Meets 27 and 28 of Oct. 1885, at Savin Rock Conn Pres. C. T. Stockwell, Springfield, Mass. Sec. A. M. Ross, Chicopee.

*Eighth District Dental Society, N. Y.*—Meets semi-annually. Next meetlng in Buffalo, April 15, 1886. Pres. S. A. Freeman; Sec. C. S. Butler, Buffalo.

*East Tennessee Dental Association*—Meets annually. Next meeting will be held at Chattanooga, Tenn., on the first Tuesday of Aug., 1886. Pres. R. A. B. Noyers, Sec. S. N. Prothro.

*New Hampshire State Dental Society*—Meets annually. Pres. H. Hill, Manchester; Sec. E. B. Davis, Concord.

*Mad River Dental Society*—Meets annually. Next meeting at Dayton, Tuesday, May 19, 1886. Pres. C. Bradley, Dayton; Sec. and Treas. L. C. Adams, Dayton,

*Mississippi Valley Dental Society*—Meets annually at Cincinnati, on first Wednesday in March, 1886. Pres. A. F. Emminger, Columbus, O.; Sec. A. G. Rose, Cincinnati.

*New England Dental Society*—Meets annually first Thursday and Friday in October, in Burlington, Vt. Pres., Jas. Lewis, Burlington Vt.; Sec., A. M. Dudley, Salem Mass.

*Northern Ohio Dental Association*—Meets annually. Next meeting at Cleveland, O. on the second Tuesday in May, 1886. Pres. D. Gale French, of Pittsburg Pa.; Rec. Sec. Geo. H. Wilson, Painesville, Cor. Sec. H. F. Harvy, Cleveland. O.

*Northwestern Dental Association* (embraces portions of Dakota, Minnesota and Manitoba) meets annually. Next meeting at Fargo, Dakota, fourth Tuesday in July, 1886. Pres. Louis Otofy, Otofy, Dak.; Sec. S. J. Hill, Fargo, Dak.

*Ohio Dental College Association*—Meets annually, Tuesday, March 2, 1886, at Cincinnati. Pres. H. M. Reid, Cincinnati; Rec. Sec. F. A. Hunter, Cincinnati.

*Odontological Society of Pennsylvania*—Meets on the first Saturday evening of each month, at 8 o'clock p. m., in the offices of the members; Pres. E. T. Darby, Rec. Sec. Ambler Tees.

*Odontological Society of New York City*—Meets the third Tuesday of each month. Pres. Wm. Jarvie; Rec. Sec. E. T. Payne.

*Pennsylvania Association of Dental Surgeons*—Pres. J. H. Githens. Rec. Sec. Theo. F. Chupein.

*Pittsburg Dental Society*—Meets the last Tuesday Evening of each month. Pres. J. B. Williams, Homestead; Sec. N. L. Reinecke.

*Fifth District Dental Society of N. Y.*—Meets semi-annually. Next meeting in Birmingham, in October. Pres. C. C. Smith, Ilion; Sec. C. J. Peters, Syracuse.

*San Francisco Dental Society*—Meets the second Monday evening of each month. Pres. W. J. Younger; Sec. W. A. Knowles; Treas. J. J. Birge.

*Sixth District Dental Society of N. Y.*—Meets semi-annually. Next meeting at Cortland, Thursday, Oct. 6th, 1886. Pres. M. D. Jewett, Richford Springs; Sec. E. D. Downs, Owego.

*St. Louis Dental Society*—Meets first Tuesday in each month. Pres. J. B. Newby; Sec. J. W. Whipple.

*Washington Dental Society*—Meets second Tuesday of each month. Pres. R. Finley Hunt; Rec. Sec. H. M. Schooly.

*Southern Dental Association*—Meets in Nashville, Tenn., on the 2d Tuesday of May, 1886. Pres. W. C. Wardlaw, Columbia, S. C.; Cor. Sec. E. S. Chisholun, Tuscaloosa, Ala.; Sec. R. A. Holliday.

*First District Dental Society of the State of New York*—Meets annually. Pres. A. L. Northrup; Sec. J. E. Dexter, New York.

---

### EXCHANGES.

St. Louis, Medical and Surgical Journal.
The Pacific Medical and Surgical Journal, San Francisco.
Cincinnati Lancet and Clinic.
Dental Cosmos, Philadelphia.
Chicago Medical Examiner.
The Detroit Review of Medicine and Pharmacy.
Medical Record, N. Y.
American Journal of Dental Science, Baltimore.
Indiana Medical Journal.
Archives of Dentistry.
The Sanitarian, New York City.
L'art Dentaire, Paris.
Le Progress Dentare, Paris.
Deutsche Monatsschrift fuer Zahnheilkunde Leipzig.—Arthur Felix.
Physician and Surgeon, Ann Arbor, Mich.
Ohio State Journal of Dental Science, Xenia, Ohio
The Microscope, Ann Arbor, Mich.
Independent Practitioner, N. Y.
The Dental Record, London.
The Journal of the British Dental Association, London.
The Southern Dental Journal.
Items of Interest, Philadelphia.
The Dental Practitioner.—The Cincinnati "Medical" News.
The New England Journal of Dentistry.
The British Journal of Dental Science.
Facts.
The Dental Advertiser.
The American Practitioner.
The Messenger of Dentistry (Russian).
The Louisville Medical News.
The Medical World.
The Peoria Medical Monthly.
The Dental Student.
American Journal of Obstetrics.
The American Pharmacist.
The Nashville Journal of Medicine and Surgery.
Science.
Medical and Surgical Reporter.
Dental Headlight.
Cincinnati Medical and Dental Journal.

### NOTICE.

We have now in stock C. Ash & Son's Teeth, all styles. Pin Teeth, 10 cents, Tube Teeth, 25 cents; Teeth without Pins, 5 cents. We also keep all shades of their Rubbers. Any one wishing to try the above can be supplied in any quantity. SAM'L A. CROCKER & CO.

### MONTHLY LIST OF PATENTS

For inventions relating to *Dentistry and Surgery* bearing date December 25, 1885. Reported expressly for this paper, by Louis Bagger & Co., Mechanical Experts and Solicitors of Patents, Washington, D. C. *Advice free.*

331,534. Manufacture of fibrous material from wood for surgical and other purposes; J. Odelga, Vienna, Austria-Hungary.
331,840. Dental suction-plate former: J. Spyer, Santa Fe, New Mexico.
332,626. Lowering mechanism for dentists' chairs; L. Stuck, Hart, Mich.
333,019. Dental chair head rest; A. R. Merrick, Blossburg, Penn.

### Second-Hand and Shopworn Dental Engines, Lathes, Etc.

WE DESIRE TO CALL ATTENTION TO THE FOLLOWING LIST OF GOODS. FOR SALE CHEAP FOR CASH.

BOXING IS NOT INCLUDED IN THESE PRICES.

### DENTAL ENGINES.

One Morrison Engine, Hodge H. P.; good order; 1 dozen burs, $22.50.

One Morrison Engine, No. 4; H. P., 1 dozen Burs, $18.00.

One Suspension Engine, good order, $25.00.

One S. S. White Water Motor Engine; good order; No. 5 hand-piece, $35.

One S. S. White Engine; 1 doz burs; good order; $28.

One Johnston Engine; good order; 1 dozen burs; $26.50.

One Morrison Improved Engine, $15.00.

One White Engine; late improved; used only short time; 1 dozen Burs, $42.50.

One S. S. White Engine; No. 4 hand piece; good order; 1 dozen burs; $30.

### DENTAL CHAIRS.

One Archer Iron Chair, No. 7; Maroon Plush, new N. P. Spittoon and Carrier, $35.00; splendid order.

One Archer No. 7 Chair and Spittoon; brown rep; good order; $25.

One Harris Chair; maroon plush; N. P. Spittoon and Carrier; fine order; $85.

One Iron Base Chair; closed arms; good order; foot stool, spittoon, and carrier; crane and table; $25.

### THE DENTAL COLLEGE

OF THE

### University of Michigan.

The Eleventh Annual Session of this Institution will commence on the 1st of October and close on the last Thursday of June, thus making a course of nine months. The regular course of instruction commences at once, and will continue through the term, with the customary vacation.

The students in this department will receive instruction in Anatomy, Physiology, Pathology, Chemistry, Materia Medica, Therapeutics, and Surgery, from the Professors of their respective branches in the *Department of Medicine and Surgery* of the University, where lectures commence, and continue the same as with the Dental College. There will also, in addition, be a special course upon Oral Pathology and Surgery, which will embrace from thirty to thirty-five lectures.

### FACULTY OF THE DENTAL DEPARTMENT.

J. B. ANGELL, LL.D. - - - - - - - - - - - President.
J. TAFT, M.D., D.D.S. Principles and Practice of Operative Dentistry.
CORYDON L. FORD, M.D., D.D.S. - - - Anatomy and Physiology.
J. A. WATLING, D.D.S. - - - Clinical and Mechanical Dentistry.
W. H. DORRANCE, DD.S. - - - - - - Prosthetic Dentistry.
J. N. Martin, M.D., - - - Lecturer on Oral Pathology and Surgery.
MARGARET HUMPHREYS, D.D.S. - Demonstrator of Clinical Dentistry.
———————— Demonstrator of Prosthetic Dentistry.

Students should be promptly present at the College on Wednesday, September 30th, at 10 o'clock, A.M., to make the preliminary arrangements for entering upon regular work. Seats in the lecture room are assigned by election to students in the order of registration on the Steward's Books; and each student is expected to occupy during the session such seat as he may select. Students, on arriving in Ann Arbor, should call at the Steward's office.

### CONDITIONS OF GRADUATION.

The candidate must be twenty-one years of age. *He must furnish satisfactory evidence of good moral character.*

He must devote three years to the study of his profession. He must attend two full courses of lectures in the Dental College, or one in some college having an equal standard of requirements, and the last one here, and we recommend that he attend three courses regularly.

He must sustain an examination satisfactory to the Faculty in all the branches taught.

A graduate of the Medical College may enter the Senior Class, and, if found qualified, may graduate after two years have been devoted to the study of dentistry.

### FEES AND EXPENSES.

The fees, which must be paid in advance, are as follows:

RESIDENTS OF MICHIGAN.—Matriculation fee, $10.00; annual dues, $25.00

NON-RESIDENTS.—Matriculation, $25.00; annual dues, $35.00.

GRADUATION FEE.—For all alike, $10.00. The admission fee is paid but once, and entitles the student to the privileges of permanent membership in any department of the University. The annual dues are paid the first year and every year thereafter while at the University.

For further particulars, address the Dean of the Dental College, Ann Arbor, Michigan.

**J. TAFT, Dean.**

Jan-86-1y

### THE DENTAL COLLEGE

OF THE

### University of Michigan.

The Eleventh Annual Session of this Institution will commence on the 1st of October and close on the last Thursday of June, thus making a course of nine months. The regular course of instruction commences at once, and will continue through the term, with the customary vacation.

The students in this department will receive instruction in Anatomy, Physiology, Pathology, Chemistry, Materia Medica, Therapeutics, and Surgery, from the Professors of their respective branches in the *Department of Medicine and Surgery* of the University, where lectures commence, and continue the same as with the Dental College. There will also, in addition, be a special course upon Oral Pathology and Surgery, which will embrace from thirty to thirty-five lectures.

### FACULTY OF THE DENTAL DEPARTMENT.

J. B. ANGELL, LL.D. - - - - - - - - - - - - President.
J. TAFT, M.D., D.D.S. Principles and Practice of Operative Dentistry.
CORYDON L. FORD, M.D., D.D.S. - - - Anatomy and Physiology.
J. A. WATLING, D.D.S. - - - Clinical and Mechanical Dentistry.
W. H. DORRANCE, DD.S. - - - - - - Prosthetic Dentistry.
J. N. Martin, M.D., - - - Lecturer on Oral Pathology and Surgery.
MARGARET HUMPHREYS, D.D.S. - Demonstrator of Clinical Dentistry.
———————————— Demonstrator of Prosthetic Dentistry.

Students should be promptly present at the College on Wednesday, September 30th, at 10 o'clock, A.M., to make the preliminary arrangements for entering upon regular work. Seats in the lecture room are assigned by election to students in the order of registration on the Steward's Books; and each student is expected to occupy during the session such seat as he may select. Students, on arriving in Ann Arbor, should call at the Steward's office.

### CONDITIONS OF GRADUATION.

The candidate must be twenty-one years of age. *He must furnish satisfactory evidence of good moral character.*

He must devote three years to the study of his profession. He must attend two full courses of lectures in the Dental College, or one in some college having an equal standard of requirements, and the last one here, and we recommend that he attend three courses regularly.

He must sustain an examination satisfactory to the Faculty in all the branches taught.

A graduate of the Medical College may enter the Senior Class, and, if found qualified, may graduate after two years have been devoted to the study of dentistry.

### FEES AND EXPENSES.

The fees, which must be paid in advance, are as follows:

RESIDENTS OF MICHIGAN.—Matriculation fee, $10.00; annual dues, $25.00

NON-RESIDENTS.—Matriculation, $25.00; annual dues, $35.00.

GRADUATION FEE.—For all alike, $10.00. The admission fee is paid but once, and entitles the student to the privileges of permanent membership in any department of the University. The annual dues are paid the first year and every year thereafter while at the University.

For further particulars, address the Dean of the Dental College, Ann Arbor, Michigan.

**J. TAFT, Dean.**

Jan-86-1y

### THE DENTAL COLLEGE

OF THE

### University of Michigan.

---

The Eleventh Annual Session of this Institution will commence on the 1st of October and close on the last Thursday of June, thus making a course of nine months. The regular course of instruction commences at once, and will continue through the term, with the customary vacation.

The students in this department will receive instruction in Anatomy, Physiology, Pathology, Chemistry, Materia Medica, Therapeutics, and Surgery, from the Professors of their respective branches in the *Department of Medicine and Surgery* of the University, where lectures commence, and continue the same as with the Dental College. There will also, in addition, be a special course upon Oral Pathology and Surgery, which will embrace from thirty to thirty-five lectures.

### FACULTY OF THE DENTAL DEPARTMENT.

**J. B. ANGELL, LL.D.** - - - - - - - - - - - - **President.**
**J. TAFT, M.D., D.D.S.** **Principles and Practice of Operative Dentistry.**
**CORYDON L. FORD, M.D., D.D.S.** - - - **Anatomy and Physiology.**
**J. A. WATLING, D.D.S.** - - - **Clinical and Mechanical Dentistry.**
**W. H. DORRANCE, DD.S.** - - - - - - - **Prosthetic Dentistry.**
**J. N. Martin, M.D.,** - - - **Lecturer on Oral Pathology and Surgery.**
**MARGARET HUMPHREYS, D.D.S.** - **Demonstrator of Clinical Dentistry.**
———————————— **Demonstrator of Prosthetic Dentistry.**

Students should be promptly present at the College on Wednesday, September 30th, at 10 o'clock, A.M., to make the preliminary arrangements for entering upon regular work. Seats in the lecture room are assigned by election to students in the order of registration on the Steward's Books; and each student is expected to occupy during the session such seat as he may select. Students, on arriving in Ann Arbor, should call at the Steward's office.

### CONDITIONS OF GRADUATION.

The candidate must be twenty-one years of age. *He must furnish satisfactory evidence of good moral character.*

He must devote three years to the study of his profession. He must attend two full courses of lectures in the Dental College, or one in some college having an equal standard of requirements, and the last one here, and we recommend that he attend three courses regularly.

He must sustain an examination satisfactory to the Faculty in all the branches taught.

A graduate of the Medical College may enter the Senior Class, and, if found qualified, may graduate after two years have been devoted to the study of dentistry.

### FEES AND EXPENSES.

The fees, which must be paid in advance, are as follows:

RESIDENTS OF MICHIGAN.—Matriculation fee, $10.00; annual dues, $25.00

NON-RESIDENTS.—Matriculation, $25.00; annual dues, $35.00.

GRADUATION FEE.—For all alike, $10.00. The admission fee is paid but once, and entitles the student to the privileges of permanent membership in any department of the University. The annual dues are paid the first year and every year thereafter while at the University.

For further particulars, address the Dean of the Dental College, Ann Arbor, Michigan.

**J. TAFT, Dean.**

Jan-86-1y

### CONTENTS.

PROCEEDINGS ........ 583

COMMUNICATIONS.

Remarks on the Prevention of Scrivener's Palsy or Writer's Cramp.... 610

SELECTIONS.

Oral Disease and Caseous Phthisis........ 616

Esmarch's Bandage in Anæsthesia........ 619

A Fungus Developed in the Human Saliva........ 620

Thinking and Working........ 620

Minutes of the Ohio State Dental Society........ 621

SALMAGUNDI ........ 626

EDITORIAL.

Obituary........ 630

---

### THE DENTAL REGISTER,

**A MONTHLY MAGAZINE DEVOTED TO THE INTERESTS OF THE**

### DENTAL PROFESSION.

*Terms Reduced to* $2.00 *Per Year. Single Copies*, 25 *Cents.*

### EDITED BY PROF. J. TAFT, D.D.S.

—AND—

### Published by SAM'L A. CROCKER & CO., Ohio Dental and Surgical Depot.

The volume commences in January and closes in December.

The REGISTER being issued monthly is always fresh, therefore Indispensable to the practitioner who desires to keep posted up with the new inventions and improvements which are daily developed. Neither expense nor effort will be spared to give value and variety to its contents. Articles will be illustrated with well executed engravings whenever they will add to the interest or assist to explanation.

Its extensive circulation and frequency of issue make it one of the BEST DENTAL ADVERTISING MEDIUMS in the United States.

All subscriptions must begin with January or July.

Local or special notices, five lines or less, will be inserted for $3 each insertion; over five lines, 50 cts. per line.

All advertising bills payable in advance, or at furthest, after the issue of the first number containing the advertisement. All transient advertisements to be paid for in advance.

☞CONTRIBUTIONS SOLICITED.

Exclusively Editorial Communications should be addressed to the Editor.

The latest number of the REGISTER may be ordered with or without other goods at any time, and all letters should be addressed to

SAM'L A. CROCKER & CO., Ohio Dental and Surgical Depot, Cincinnati, O.

